# Supplementary material for: Fieldoscopy at the quantum limit
Source: Light Sci Appl. 2026 Feb 9;15:110. doi: 10.1038/s41377-025-02066-8 (PMC12886900; doi:10.1038/s41377-025-02066-8)
Supplement: Supplementary file 1 — Supplementary materials for fieldoscopy at the quantum limit [file 41377_2025_2066_MOESM1_ESM.docx]

Supplementary Information for

**fieldoscopy at the quantum limit**

Dmitry A. Zimin^1,2*^, Arjun Ashoka^1^, Florentin Reiter^3,4^ and Akshay Rao^1^

*^1^Cavendish Laboratory, Department of Physics, Cambridge University, CB3 0HF Cambridge, United Kingdom*

*^2^Laboratory of Physical Chemistry, ETH Zürich, Vladimir-Prelog-Weg 2, 8049, Zürich, Switzerland*

*^3^Institute for Quantum Electronics, ETH Zürich, 8093 Zürich, Switzerland*

*^4^Quantum Center, ETH Zürich, 8093, Zürich, Switzerland*

*^*^Corresponding author: dzimin@ethz.ch*

**S1 Laser beamlines**

The laser beamline for experiments was a commercial tabletop mode-locked Yb-based laser (Pharos, light conversion). The details on the experimental beamline with CEP-stabilized pulses can be found in [1, 2].

**S2 Optical schematics of experimental setups**

The schematics of the experimental setups for CEP-stabilized and CEP-unstabilized measurements are shown on Figs. S1 and S2. For CEP-stabilized detection, the nonlinear medium was z-cut α-quartz crystal of ∼ 12 μm thickness [1]. For CEP-unstabilized detection, the nonlinear medium was 1 mm thick Barium Borate (BBO). The wedge pairs WP1 and WP2 were used to fine-tune the CEP in each optical arm.

**S3 Detection frequency**

In both, CEP-stabilized and CEP-unstabilized experiments, the detection frequency was defined by the bandpass filter placed after the nonlinear crystal. In the CEP-stabilized case with a central wavelength of ~ 750 nm, the narrow bandpass filter centered at 355 nm (Thorlabs) was used. In the CEP-unstabilized case with 1030 nm central wavelength, the narrow bandpass filter center at 515 nm (Thorlabs) was used.

**S4 Data acquisition**

In CEP-stabilized experiments, the signal was a current generated by a silicon photodiode. This current was first converted to voltage with further amplification by a transimpedance amplifier (DLPCA-200, FEMTO Messtechnik). The amplified voltage signal was then connected to a dual-phase lock-in amplifier (SR-830, Stanford Research Systems), triggered by an electrical signal synchronized with a half of the repetition rate of the laser. The measured signal from the lock-in amplifier was read by software on a computer via GPIB interface (National Instruments).

In CEP-unstabilized experiments with a laser oscillator, the signal was a current generated by a silicon photodiode. This current was first converted to voltage with further amplification by a transimpedance amplifier (DLPCA-200, FEMTO Messtechnik). The amplified voltage signal was then connected to a dual-phase lock-in amplifier (Zurich instruments), triggered by an electrical signal synchronized with a chopper installed in the test arm. The measured signal from the lock-in amplifier was read by software on a computer via USB interface.

**S5 Fieldoscopy versus intensity-based photon detection**

In contrast to intensity-based methods, fieldoscopy measures a signal proportional to the electric field, which scales with the square root of the number of incoming photons. This distinction has important implications. When detecting light intensity or photon counts, reducing the photon number by a factor of 2—such as by blocking every second pulse—leads to a corresponding halving of the signal. However, when detecting the electric field, halving the photon number decreases the signal only by a factor of $\sqrt{2}$, which is not equivalent to blocking alternate pulses. Fig. S3 illustrates this contrast by comparing intensity and electric field detections in the regime of perfectly coherent states governed by Poisson statistics. In the simulation, we analyze how the signal power and its standard deviation scale with the mean photon number ⟨n⟩. Overall, the results in Fig. S3 show that intensity measurements alone do not reveal the transition from classical to quantum light, in contrast to the electric field.

**S6 Fieldoscopy in the yoctojoule regime**

The fieldoscopy measurement of the test field with a mean pulse energy in the yoctojoule regime was performed identically as the measurements with higher pulse energies. The test pulse was attenuated with a set of three natural density filters and wire grid polarizers for fine tuning of the pulse energy. The transmission of the natural density filters was characterized by measuring the incident and transmitted powers of the laser beam consisting of many photons. After the transmission of the laser pulse through the set of natural density filters and wire grid polarizers, the measured average pulse energy was 876 yocto joules which corresponds to the 0.0045 mean photon number $\left\langle n \right\rangle$. The results of the fieldoscopic measurement are shown in Fig. S4. We note that in the regime of $\left\langle n \right\rangle$ = 0.0045, considering perfectly coherent state described by the Poisson distribution, 99.55 % of pulse are in the vacuum state (no photons). Out of the remaining 0.45 % of pulses that contain photons, only 0.22 % consist of more than one photon and 99.78 % of pulses are in a single photon state. This statistical distribution leads to a very irregular signal (Fig. S4a). However, by taking a Fourier transformation of the measured trace shown in Fig. S4a, the signal around 0.29 PHz (1030 nm) frequency (Fig. S4c) is clearly observed. Similarly to the measurements at higher pulse energies we also record a standard deviation for each temporal delay between sampling and test pulses (Fig. S4b). In the absence of the test field, the standard deviation remains constant. In the presence of the test electric field, we expect a standard deviation to follow an envelope profile of the field. We indeed observe that the measured standard deviation is increasing as the temporal delay approaches 0 fs, and then decreases. This trend follows the envelope profile of the test field similarly to the measurements with larger photon numbers. Lastly, since the standard deviation is proportional to the absolute value of the electric field, in the frequency domain of the recorded temporal standard deviation, we expect spectral components around second harmonic (0.58 PHz) of the fundamental test pulse frequency (0.29 PHz). Indeed, Fig. S4d shows a distinct peak around 0.58 PHz.

Overall, our measurement demonstrates that in the extreme regime of weak coherent states with $\left\langle n \right\rangle$ = 0.0045, the measurement becomes very noisy due to irregularity of the photon arrivals. However, even in this regime, the distinct signatures of the test field are clearly observed.

## References

1. Zimin, D. A., Yakovlev, V. S. & Karpowicz, N. Ultra-broadband all-optical sampling of optical waveforms. Sci. Adv. 8, (2022).
2. Sederberg, S. et al. Attosecond optoelectronic field measurement in solids. Nat Commun 11, (2020).

## Supplementary figure legends


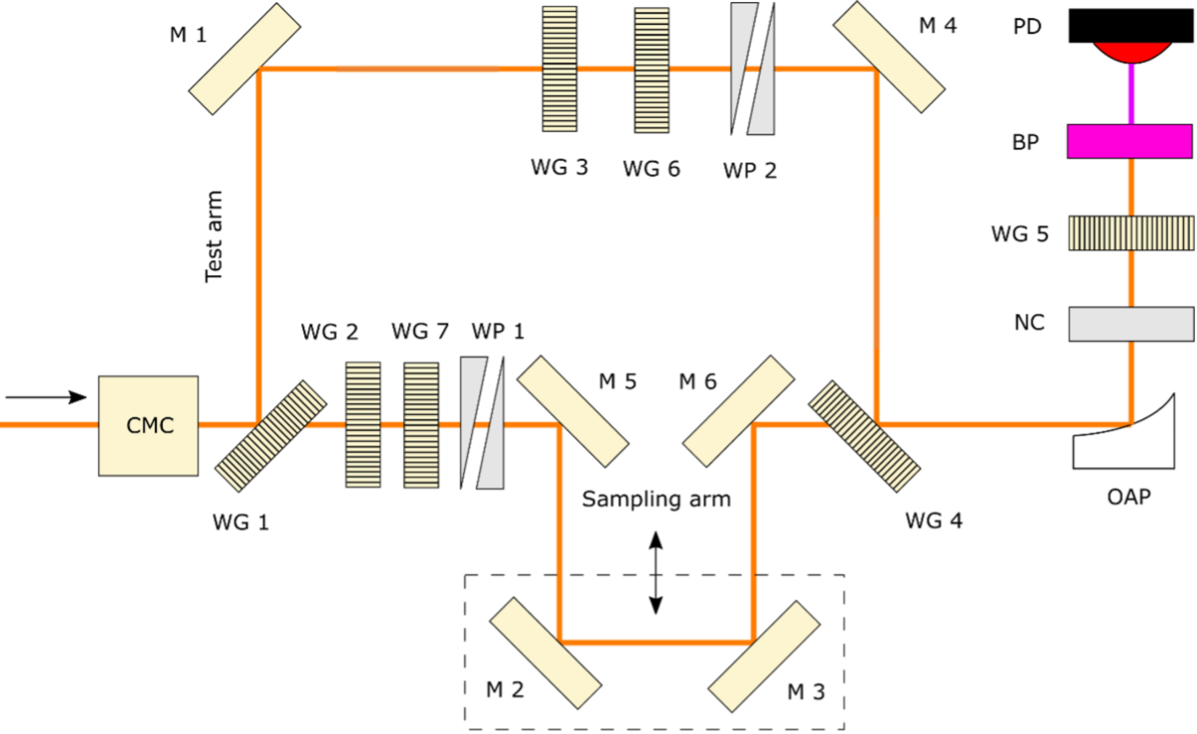


**Fig. S1 | Optical setup for CEP-stabilized experiments.** WG – wire-grid polarizer, WP – wedge pair, M – protected silver mirror, NC – nonlinear crystal, BP – bandpass filter, PD – photodiode, CMC – chirped mirror compressor, OAP – off-axis parabolic mirror.


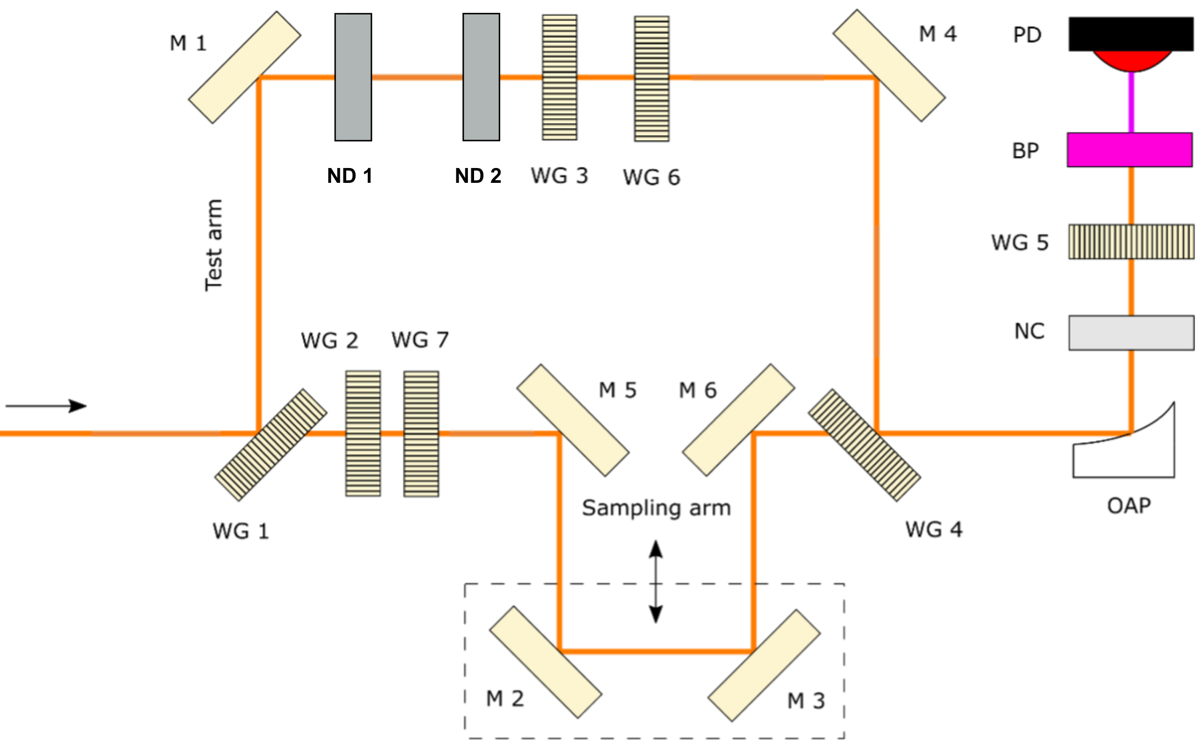


**Fig. S2 | Optical setup for CEP-unstabilized experiments with a laser oscillator.** WG – wire-grid polarizer, M – protected silver mirror, NC – nonlinear crystal, BP – bandpass filter, PD – photodiode, OAP – off-axis parabolic mirror, ND – natural density filter.


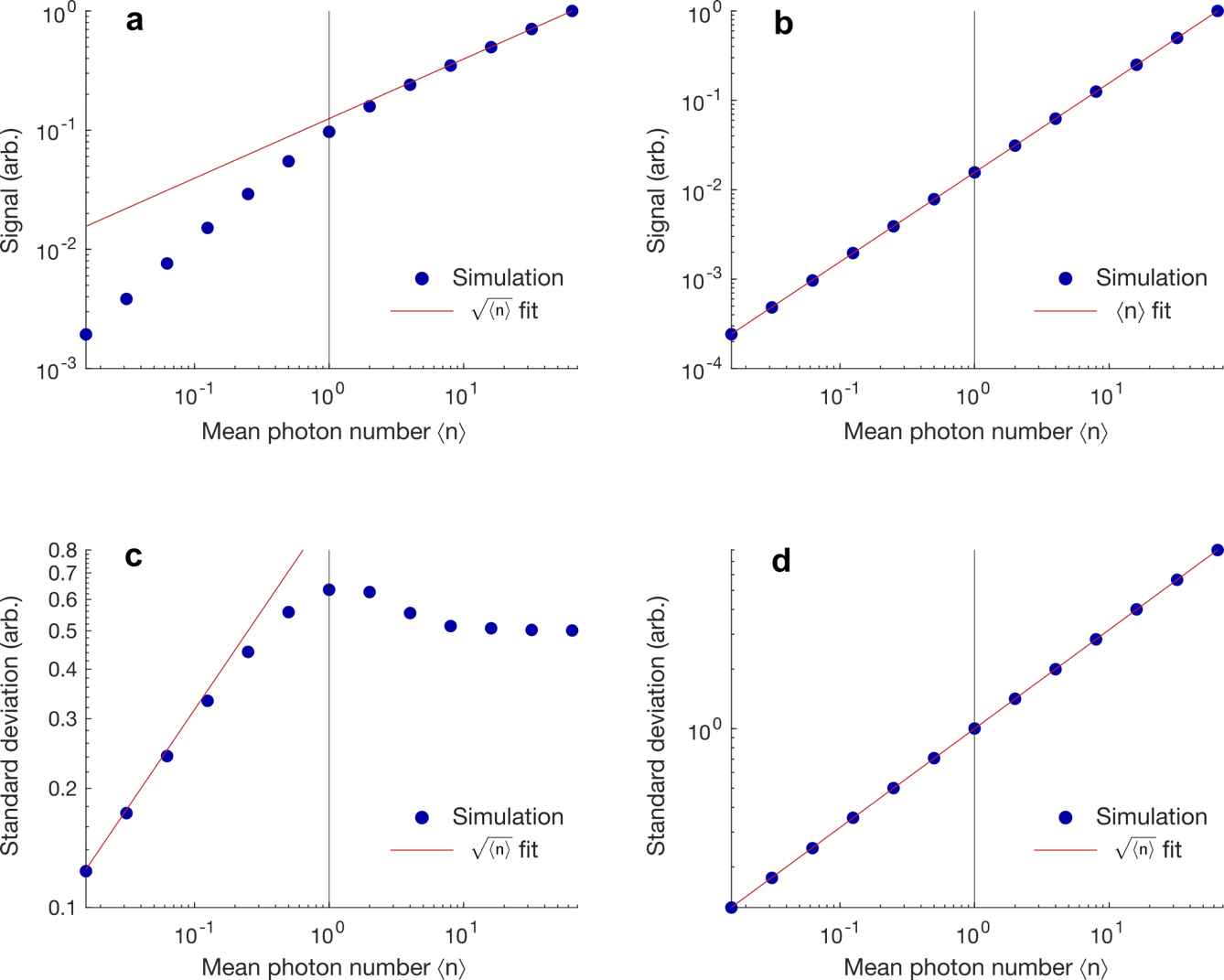


**Fig. S3 | Fieldoscopy vs spectroscopy.** **a**, Simulated field scaling vs mean photon number. **b**, Simulated intensity scaling vs mean photon number. **c**, Simulated scaling of the field standard deviation vs mean photon number. **d**, Simulated scaling of the intensity standard deviation vs mean photon number.


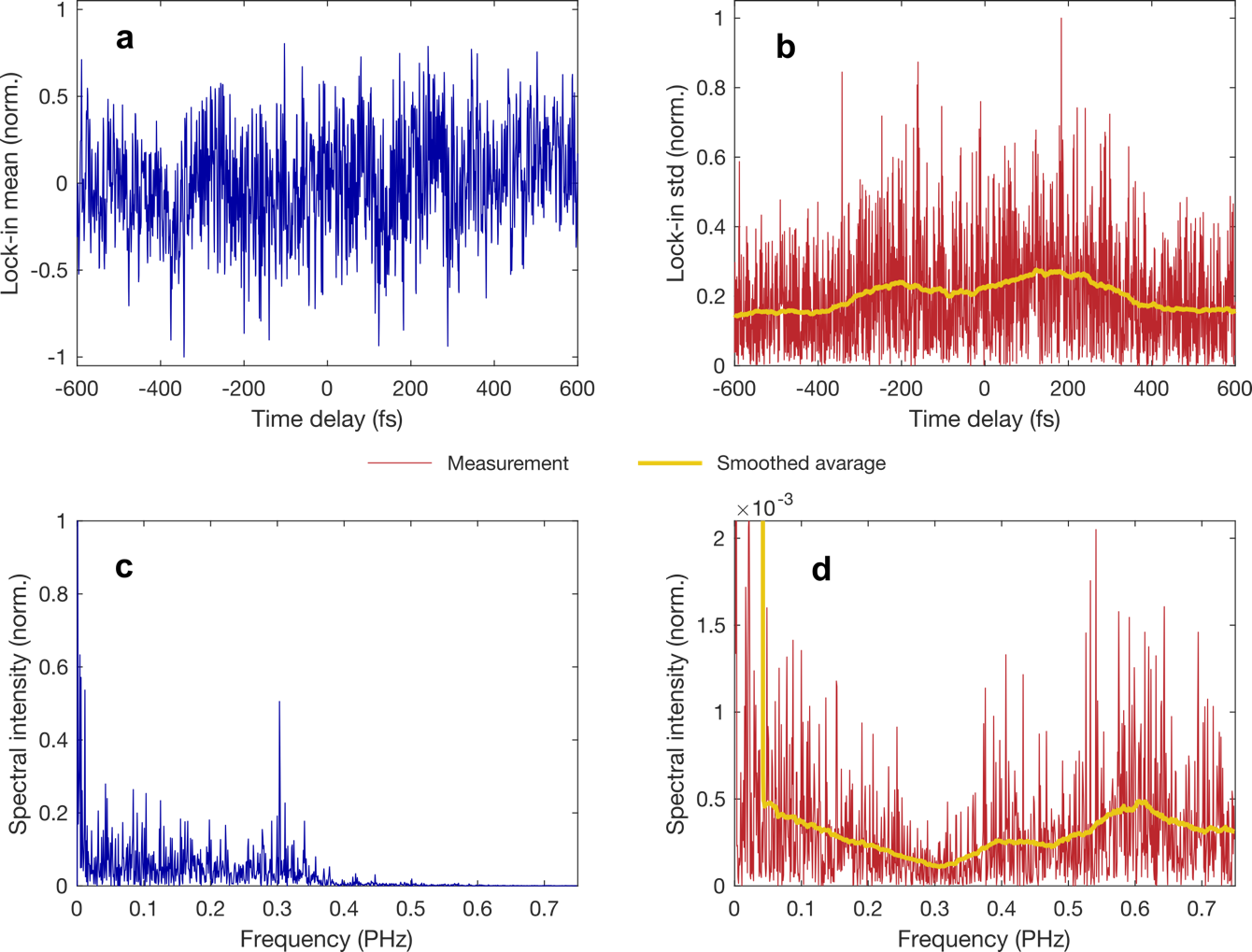


**Fig. S4 | Yoctojoule-level fieldoscopy.** **a**, Measured lock-in signal versus the time delay between sampling and test pulses. **b**, Measured standard deviation for each temporal delay in **a**. **c**, Spectrum of the measured signal in **a**. **d**, Spectrum of the measured standard deviation in **b**. The yellow curve represents a smoothed moving average of 200 points.
